# 

**Published:** 1898-09-24

**Authors:** 


### NOTICE TO CORRESPONDENTS.

**All MS., Letters, Books for Review, and other matters intended for the Editor should be addressed The Editor, "The Hospital," The Hospital Building, 28 & 29, Southampton Street, Strand, W.C.**

**The Editor cannot undertake to return rejected MS., even when accompanied by stamped directed envelope.**

**Notice to Advertisers and Subscribers.**

**All Advertisements, Orders for Copies of The Hospital, and Business Communications should be addressed to THE MANAGER, (Not to The Editor), The Hospital, The Hospital Building, 28 & 29, Southampton Street, Strand, London, W.C.**

**The Cost of Subscription, post free, is as follows:—Per week, 2½d. per quarter, 2s. 8d.; per half-year, 5s. 3d.; per annum, 10s. 6d. Foreign:—Per annum, 15s. 2d.; payable, in all cases, in advance.**

### Scraps and Gleanings.

Great endeavours are being made to render the bazaar in aid of the Aberdeen Children's Hospital a success.

The work of extension at the Dundee Royal Infirmary will shortly be commenced. The extension of the convalescent home is progressing rapidly.

A cheque for £2,695 odd, the balance of the Warneford Hospital Jubilee Fund, has been handed to the Secretary of the Warneford Hospital.

The new operating theatre of the Swansea Hospital, which is the gift of Mr. Ben Evans, is to be opened by Sir W. MacCormac on October 28th.

The Sheffield Infirmary is appealing for the sum of £10,000 required to erect a new operating theatre, and to pay off the debt of £5,000 on the Nurses' Home.

Some difficulty is being experienced in filling the vacant chairmanship at Swansea Hospital, two or three gentlemen who have been approached all declining the position.

